# Images of Appalachia

**Published:** 2006-09-15

**Authors:** Eugene J Lengerich, James R Bohland, Pamela K Brown, Mark B Dignan, Electra D Paskett, Nancy E Schoenberg, Stephen W Wyatt

**Affiliations:** The Pennsylvania State University, Health Evaluation Sciences; Institute for Community Health, Virginia Polytechnic Institute and State University, Blacksburg, Va; Mary Babb Randolph Cancer Center, West Virginia University, Morgantown, WVa; Markey Cancer Center, Department of Internal Medicine, University of Kentucky, Lexington, Ky; Comprehensive Cancer Center, School of Public Health, The Ohio State University, Columbus, Ohio; Department of Behavioral Science; College of Public Health, University of Kentucky, Lexington, Ky

The theme of this issue of *Preventing Chronic Disease* *(PCD)* — Appalachia — will stir a collage of images in readers' minds. A dominant image is likely to be derived from the great Appalachian mountain chain of eastern North America. A saddle gap in the top ridge of that mountain chain may yield a glimpse of a meandering river whose banks are covered with pastures and small towns where friends and family make their own traditional music. Another image may be of the great oaks, strong hickories, and straight tulip poplars of the mountainside. Along seeps and creeks, the shadow cast by an overarching canopy of hemlock boughs provides relaxation and restoration to a wayfaring hiker.

The natural features of mountain ridges, forests, and riverbanks are the basic elements of many images of Appalachian cities, townships, and boroughs. In the bends of great rivers, an Appalachian city might be dominated by a steel mill or paper plant. Dairy and beef farms create a pastoral landscape in Appalachia's townships and counties. Coal mines in the Appalachian mountainsides have been the livelihood of many families for generations.

Another dominant image of Appalachia is that of tight-knit communities composed of self-reliant residents with a distinct cultural heritage. Faced with limited economic opportunities and, for some, pervasive poverty, Appalachian communities remain vibrant. The Appalachian community itself may be a substantial source of its residents' strength. Although the recent disasters in the coal mines of Appalachian Kentucky, Pennsylvania, and West Virginia have been devastating and tragic, they have also given America front-page images of residents rallying around lifetime members of the community, ringing bells from church steeples for breaking news, and offering camaraderie both above and below the ground.

To epidemiologists and public health researchers, the predominant images of Appalachia are those of increased chronic disease burden, limited access to health care, and elevated rates of behavioral risks. Surveillance data collected over decades show that this image is generally correct. Among whites in Appalachia, coronary heart disease mortality increased from 1980 to 1993 (1). Lung, colorectal, and cervical cancer mortality (2) and incidence (3) were found to be elevated in Appalachia. Residents of Appalachia have increased behavioral risks (4). The National Cancer Institute confirmed that mortality rates from cervical cancer were especially high in Appalachia and concluded that cervical cancer mortality is a marker for larger health system concerns, including medical care access, cultural issues, and health communication and education (5). In this issue of *PCD*, Wewers et al (6) report on the prevention of risk factors for chronic disease, including tobacco use, energy imbalance, and sexual behavior, in Appalachian Ohio.

The heterogeneous nature of communities in Appalachia's broad geographic area suggests that a community-based, participatory approach to reducing the burden of chronic disease is advisable, possibly essential. Appalachian communities are as unique as the trees on an Appalachian mountainside; they should be engaged in defining the public health problem, formulating an action plan, and evaluating the outcome. In this issue, Kluhsman et al (7) report on a network of community cancer coalitions in Appalachia, Coyne et al (8) provide a unique glimpse into the social and cultural characteristics of Appalachian communities, and Tessaro et al (9) and Lyttle et al (10) offer perspectives on early detection of cancer and cancer screening in Appalachia.

Three broad recommendations arise from this themed issue. First, because Appalachia is a clearly defined region of the United States, a regional approach to public health surveillance is needed to support collection and analysis of health-related data. The federal government and professional organizations should consider adopting specific efforts to increase disease and risk factor surveillance in Appalachia. Second, interventions to reduce chronic disease should be community-based and participatory because of distinct, heterogeneous communities in Appalachia. Appalachian communities, with their strong history and cultural heritage, have demonstrated the potential and need for a community-based and participatory approach.

Finally, public health practitioners need evidence-based approaches that have been developed or evaluated with the characteristics of the Appalachian population in mind; unfortunately, few exist. Consequently, practitioners in Appalachia are frequently asked to adapt evidence-based programs that have been created for other populations to Appalachian communities; however, adaptations of programs tested among populations defined by race or ethnicity are at risk of reducing program fidelity.

Partnerships of researchers, community members, and health care providers offer a reasonable mechanism to address these recommendations for Appalachia. Together, these partnerships would be equipped with the experience and community involvement necessary to begin to address some of this Appalachian disparity. Concerns related to cancer education, research, and training are being addressed by the Appalachian Community Cancer Network, which includes partners in Kentucky, Maryland, New York, Ohio, Pennsylvania, Virginia, and West Virginia (11).

If these recommendations were implemented fully, the future image of disease prevention and health promotion in picturesque Appalachia would indeed be brighter. At that time, the drop in the rates of chronic disease in Appalachia, like the gaps in the mountain ridges of the region, will offer glimpses of spires of success in those communities and towns.
